# The Welfare of Beef Cattle in the Scientific Literature From 1990 to 2019: A Text Mining Approach

**DOI:** 10.3389/fvets.2020.588749

**Published:** 2021-01-11

**Authors:** Elena Nalon, Barbara Contiero, Flaviana Gottardo, Giulio Cozzi

**Affiliations:** ^1^Eurogroup for Animals, Brussels, Belgium; ^2^Department of Animal Medicine, Production and Health (MAPS), University of Padua, Padua, Italy

**Keywords:** beef cattle, animal welfare, text mining, topic analysis, research

## Abstract

Beef cattle are the third most numerous terrestrial farmed animals worldwide. Factors such as geographical region, animal category, breed, and rearing system pose specific animal welfare challenges that can have an impact on animal and public health. This article uses text mining (TM) and topic analysis (TA) to explore the scientific literature on beef cattle welfare published in English from 1990 to 2019. Our aim was to reveal the main research topics and their evolution over time. Our analysis showed that the three most relevant themes in research since 1990 have to do with calf behaviour and management, efficiency, and environmental sustainability, and the effects of transport and slaughter on meat quality. Topics showing the most marked increase in the number of papers published deal with stakeholders' perceptions and market opportunities for added-value beef products and risk factors for morbidity and mortality, especially in relation to calf health, antimicrobial use, and antimicrobial resistance. The results indicate a particular focus on the welfare of calves, especially in the veal industry. Pain relief during the castration of calves and bulls also featured prominently. Research is also increasingly assessing aspects of beef cattle welfare that are interlinked to meat quality, the social and environmental sustainability of the sector in relation to market opportunities, and public health. The identified topics represent a basic source of information that can be used for further and more detailed analyses (e.g., systematic reviews) focussed on specific research themes or geographical areas.

## Introduction

Beef cattle (including buffaloes) are the third most numerous farmed animals worldwide (after poultry and pigs), with 71.61 million tonnes produced globally in 2018. In the same year, the EU produced 10.64 million tonnes of beef meat (1). Globally, cattle meat production has more than doubled since 1961, increasing from 28 million tonnes per year to 68 million tonnes in 2014 (1). In numerical terms, between 2000 and 2050 the global cattle population might grow from 1.5 billion in 2000 to 2.6 billion (2). Beef cattle farming practices differ substantially across the world, ranging from extensive to intensive and using different breeds (3). Each rearing system presents specific challenges for animal welfare (4–6) and, while guidance on best practice is available for some of the identified problems, knowledge gaps persist, for instance in the areas of disease prevention and monitoring, optimisation of live transport, use of environmental features, and enrichments (4). Specific issues are associated to the management, transport and rearing of male calves coming from the dairy industry, which are of limited commercial value (7).

The science of animal welfare has considerably evolved from the 1990s to the present day, and with it the recognition that animals are sentient beings, deserving of “a good life,” which includes opportunities to experience positive affective states (8). It is therefore of interest to investigate if and under what respects the sustained global increase in cattle production has propelled an interest in researching beef cattle welfare. This paper uses text mining (TM) and topic analysis (TA) to analyse the scientific literature on beef cattle welfare published in English from 1990 until 2019 to better understand the most important topics discussed in academic publications and their evolution over time. TM is defined as “The knowledge discovery process which looks for identifying and analysing useful information on data which is interesting to users from big amounts of textual data” and as such it is unique in its ability to analyse concept relationships “in order to find new structures, patterns or associations” and to “discover new facts and trends about the world itself” (9). More in detail, TM can be used to summarise or cluster information into charts or maps; identify hidden structures (and associations) between concepts or groups or concepts; extract hidden associations between textual elements; provide an overview of the contents of a large collection of documents; categorise texts by discovering relevant groupings (9). In other words, TM and TA represent tools that can produce a preliminary thematic screening of large numbers of documents to reveal a structured “map” of textual knowledge (10, 11) by uncovering recurrent topics and latent themes when the set of documents to analyse is large. For these reasons, TM is increasingly being used in the scientific literature as a tool to identify themes and future research avenues across a broad range of topics, including animal welfare studies (12, 13).

## Materials and Methods

### Data Set

A literature search protocol was set up to identify peer-reviewed papers with at least an English abstract that covered the topic of beef cattle welfare using Scopus®, the abstracts and citation database of Elsevier^©^. The keywords used were “bovine meat welfare,” “meat cattle welfare,” “veal welfare,” “beef welfare,” “beef cattle welfare,” and “heifer welfare.” The search was performed in January 2020. The initial timespan considered was between 1960 and 2019. As less than one relevant paper per year was published in the period 1960–1989, only papers published from 1990 onwards were retained for the full statistical analysis. An electronic Excel workbook was used to collect the data extracted from the identified papers. The spreadsheet was built in a 2-way table format considering every paper (record) as a row and its descriptive information in columns. A list of the descriptors used and additional information on data format are provided as Supplementary Materials. All datasets were merged and overlapping records were erased. Reasons for automatic exclusion were: no author available, no source available, document type erratum, no document type available. Additionally, two reviewers (EN and BC) independently screened the titles and abstracts for relevance to the research topic (i.e., papers dealing with one or more aspects of beef cattle welfare). The criteria for manual exclusion of the papers were (1) wrong topic or focus (for instance, social and economic welfare) or (2) wrong species or sector (e.g., welfare of dairy cattle). Citations were excluded from the database if one or both criteria for exclusion were chosen by both reviewers for the same paper. Disagreements were resolved by consensus with the mediation of FG. The geographical localisation of each record was derived based on the affiliation of the corresponding author/first author. Some descriptive statistics of the selected records were prepared to profile the scientific corpus based on information recorded from the Scopus database. A regression analysis of the number of published papers on years was performed to calculate the trend by year of the scientific interest for this topic.

### Text Mining

A TM analysis was performed on the abstracts of the selected papers to find important patterns in text data as described by Wang et al. (10) and Contiero et al. (12). This technique converts text into numeric information and highlights the word frequency distributions. The text pre-processing consisted in three steps: tokenisation, filtering and stemming (14). Tokenisation is the process of finding words, separating them and reducing them to lowercase. Filtering is also called stop-word removal (exclusion of characters such as punctuation and blanks, exclusion of stop words such as articles, prepositions, and conjunctions, etc.). Stemming reduces word variants to their root form and we used Porter stemming algorithm to perform this feature (15). In addition, keywords used in the bibliographic search were removed to avoid poor discriminative information due to their presence in almost all abstracts retrieved (10). The words were organised into a matrix that contains the documents along the rows and the terms along the columns (so-called document-term matrix). A term frequency-inverse document frequency technique (TFIDF) was used to attribute a relative weight to words (16). This represents the frequency of a term adjusted for how widely it is used, thus reflecting how important a word is in the whole collection of documents. The words with the greatest relevance (TFIDF ≥ 8) were represented as histogram. A cloud of the most relevant words (TFIDF≥5) was also created (https://www.wordclouds.com/) in which a bigger character size indicates a higher TF-IDF value. The statistical analysis was conducted with R package (2017) using the libraries tm, stringr, and SnowballC.

### Topic Modelling

Topic modelling is a tool to uncover the structure of meaningful themes among collections of documents as well as to discover hidden textual patterns [something similar to a principal component analysis of a given dataset of words; (17)]. Latent Dirichlet allocation (LDA), one of the most popular approaches to perform topic modelling analysis, was applied for the text mining of our abstract corpus (10). A single topic can be described as a multinomial distribution of words, and a single document can be described as a multinomial distribution of latent topics. This model provides both a topic representation of all the documents and the word distributions of all the topics, in an iterative process implemented using a Gibbs sampling. At the end of the iterative process, a posterior distribution was calculated to estimate the topic mixture of each document (by counting the proportion of words assigned to each topic within that document) and the words associated to each topic (by counting the proportion of words assigned to each topic overall). We used LDA function with Gibbs sampling option of the *topicmodels* package in R (18). The individual topics were presented as an unstructured set of words using the bar histogram representation, where every bar relative to every word is proportional to the probability of the word within a topic (beta value). The cumulative probability of the 10 most probable words for different numbers of topics was calculated.

The number of topics needs to be fixed a-priori. As the “ideal” number is in general not known, several models with different number of topics were fitted and measures of evaluation were calculated. In a first approach, the perplexity of holdout and training datasets was calculated. Perplexity measures how well a probability model predicts a sample. A lower perplexity score indicates better generalization performance (19). The document-term matrix was split in two parts: the first one, which included 80% of the documents, was used as training dataset and the last one as test dataset (hold-out set). For different numbers of topics (from 2 to 20) LDA models were fitted on the training dataset. Using the results obtained in the training dataset, the perplexity index was calculated both for the training and the holdout datasets. This machine learning approach permits to test the adequacy of a model developed in a training dataset measuring its performance on an hold-out dataset. A second approach to fix the number of topics is based on the harmonic mean of the likelihood of a set of samples generated by the Gibbs sampler (20). In this case a higher value of the harmonic mean is better. A hierarchical cluster analysis approach was adopted for the topics analyses with different number of topics. The topic-word matrix (first 100 most probable words) was transformed to binary data with a 1/0 to indicate presence of a word in a given topic. Finally, a trend analysis of the proportion of each topic by year was performed to test the dynamics of all topics over time.

To explore the relationship between topics, we performed hierarchical clustering analysis. The results are presented as Supplementary Materials.

## Results

### Descriptive Statistics

The distribution of the results of the initial bibliographic search by string on titles, abstracts, and keywords is shown in Table 1. The most numerous articles concerned the string “beef welfare” (81%), followed by “beef cattle welfare” (60%), “meat cattle welfare” (41%), “heifer welfare” (30%), “bovine meat welfare” (15%) and, lastly, “veal welfare” (11%). After removal of overlapping records and manual removal of irrelevant ones, 983 records were retained for further analysis.

**Table 1:** Bibliographic search strings for the text mining analysis on the welfare of beef cattle carried out on titles, abstracts and keywords of peer-reviewed literature in English published between 1990 and 2019.

**Search string**	**Original n. of records**
Beef welfare	794
Beef cattle welfare	594
Meat cattle welfare	412
Heifer welfare	295
Bovine meat welfare	147
Veal welfare	110

**Figure 1:**
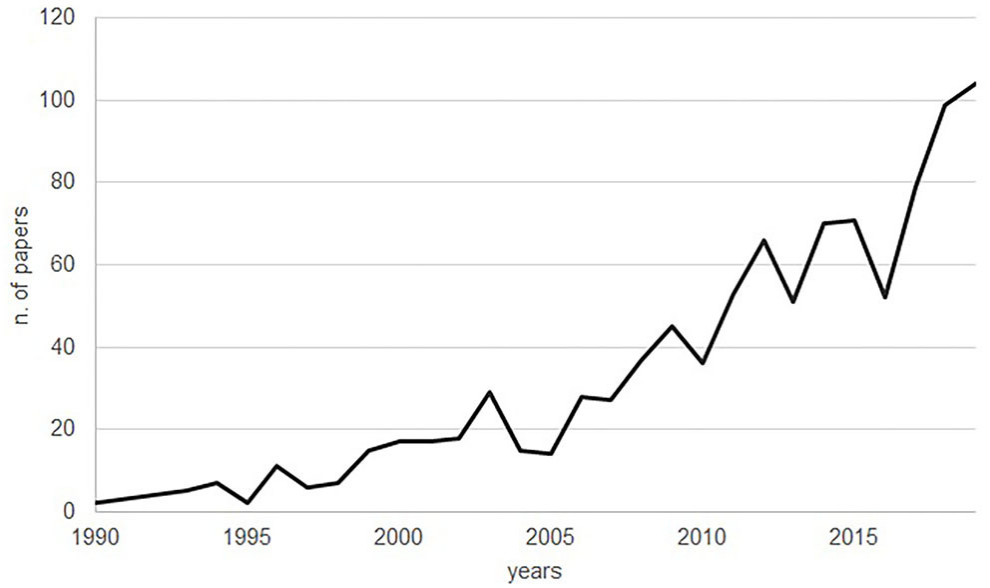
Peer-reviewed articles on the welfare of beef cattle published within the period 1990–2019. From 1996 onwards there was a significant increase in the number of papers published annually (by interpolation: 3/year).

Looking at the trend for the number of papers per publication year, from 1990 to 1996 fewer than 10 papers on beef cattle welfare were published annually, whereas from 1996 to 2019 there was a significant increase of 3 papers per year (interpolation of data using a regression analysis from 1990 to 2019: y = 3.28x-6529 *R*^2^=0.85; Figure 1).

Nearly half of the identified papers had first or corresponding authors based in Europe (members of the European Economic Area and Switzerland; 46%). The second most important area of provenance of authors was North America (25%), followed by South America (11%), Oceania (9%), Asia (6%) and, lastly, Africa (3%).

**Figure 2:**
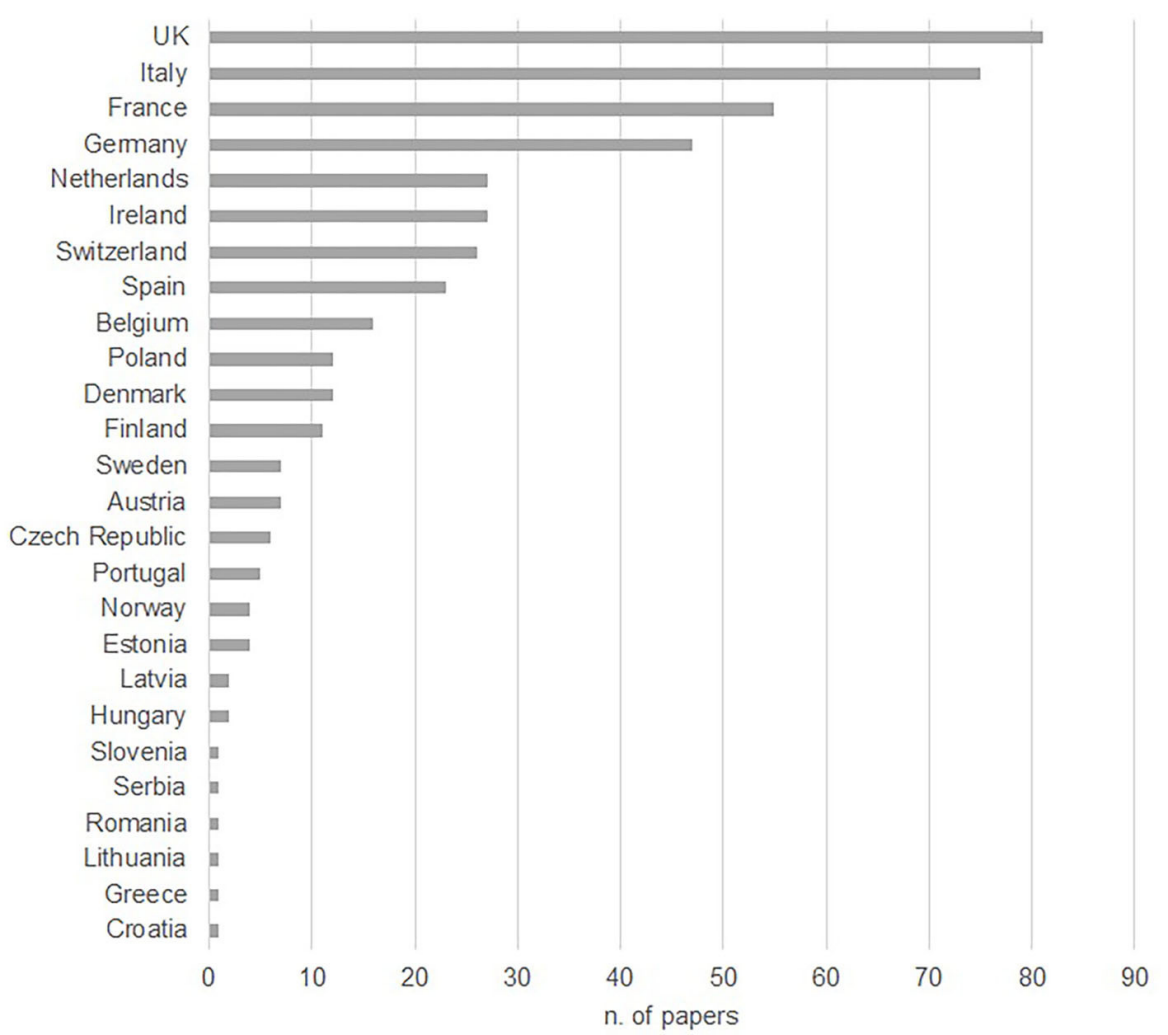
Distribution by European country of peer-reviewed papers on the welfare of beef cattle based on the nationality of the first/corresponding authors and published between 1990 and 2019.

A breakdown of articles per country of the European block is shown in Figure 2.

**Figure 3:**
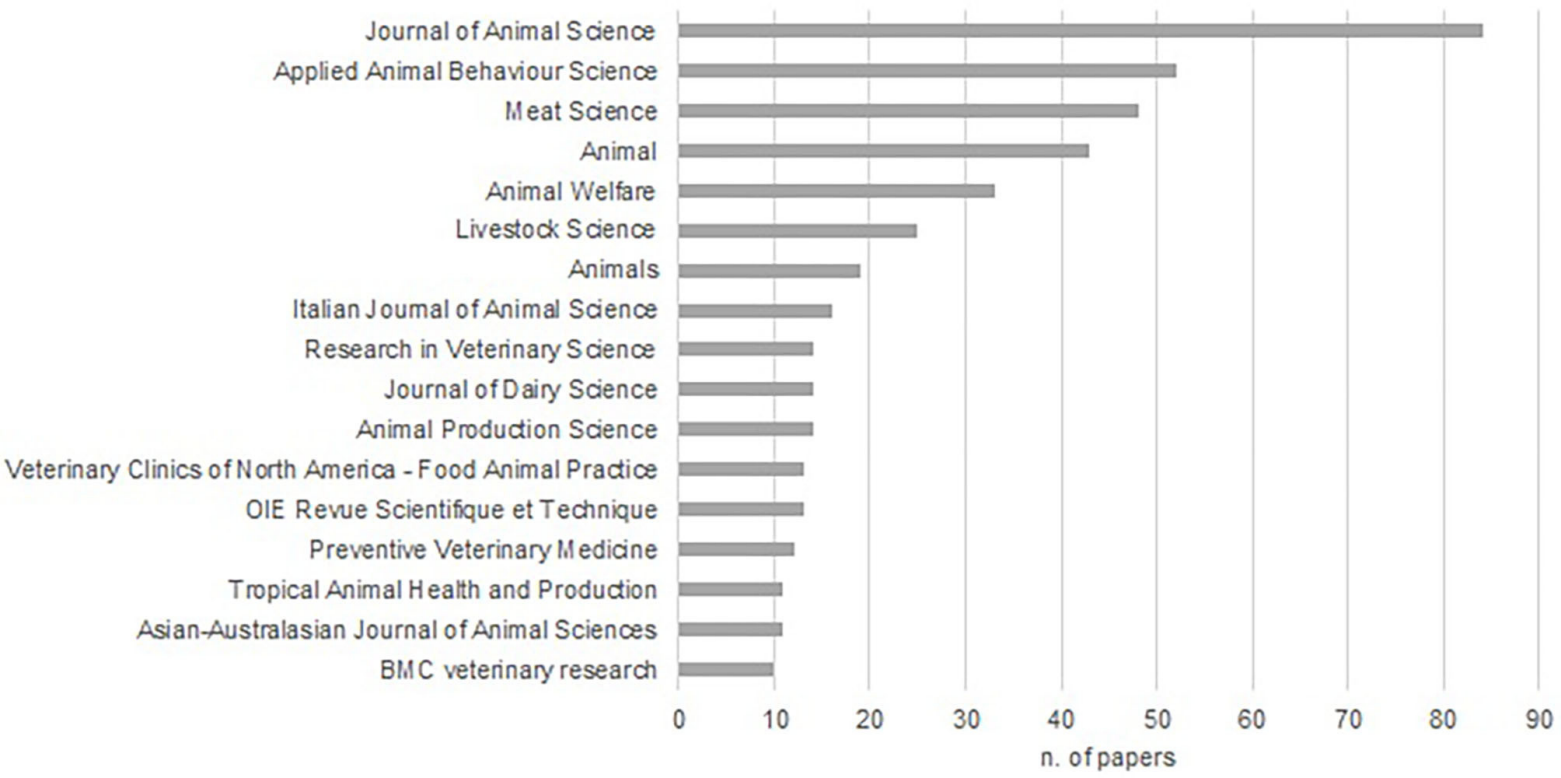
Distribution of published papers by journal title (with at least 10 papers published on the topic in the period considered). The most frequent publishing sources of the retained papers were scientific journals dealing with animal (production) science, animal welfare and behaviour, and meat quality, whereas the journals focused on veterinary science were a less important publishing channel (15%).

The distribution of published papers by journal title (with at least 10 papers published on the topic in the period considered) is shown in Figure 3. The most frequent publishing sources of the retained papers were scientific journals dealing with animal (production) science, animal welfare and behaviour, and meat quality, whereas the journals focused on veterinary science were a less important publishing channel (15%).

Research articles represented the most common type of retained paper (76%) followed by reviews (13%), conference papers (6%), and others (5%). The papers were published in 310 scientific journals. As a whole, the 983 articles had collected—as of January 2020—a total of 15,208 citations. The most cited article was published in 2004, collecting 392 citations, followed by an article published in 2010 that collected 235 citations.

### Text Mining Results

**Figure 4:**
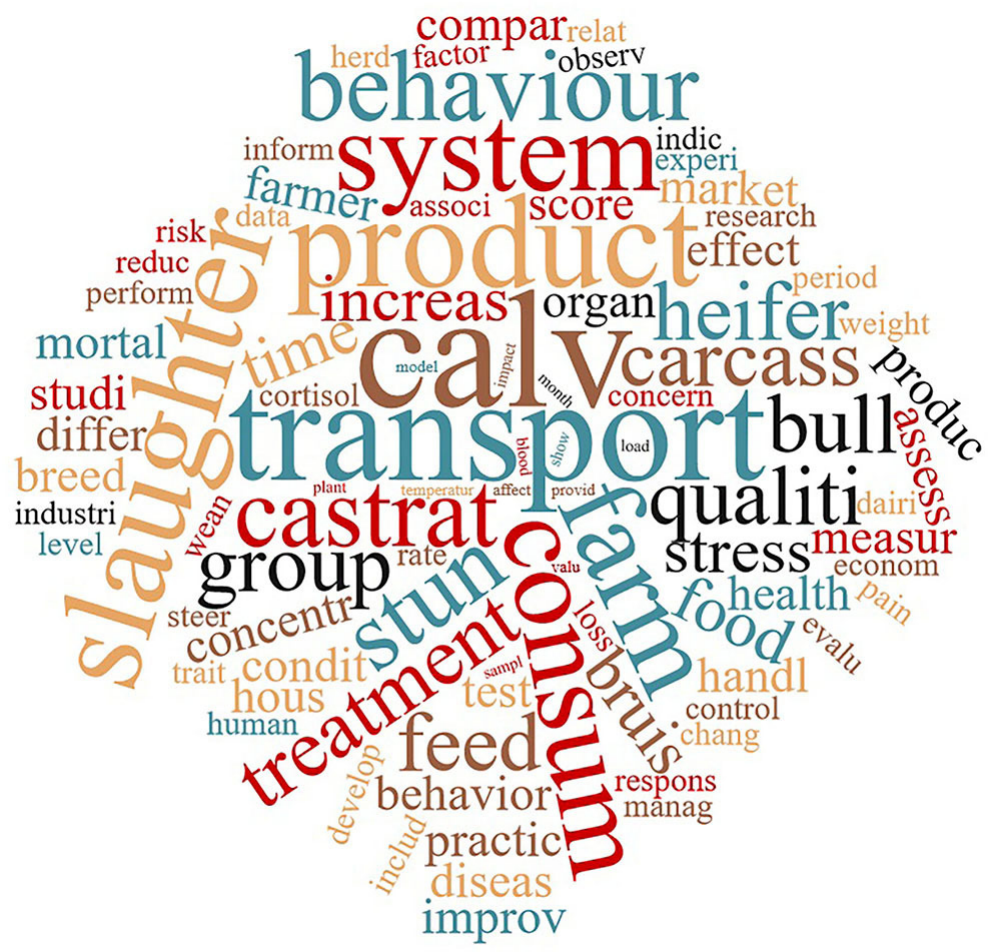
Cloud representation of the most relevant words (stems) in the database (TFIDF≥5). The relative importance of the terms is reflected by their size.

Text mining analysis was performed to identify the most important words of the data corpus. The pre-processing of the data produced 106,108 words and after reduction of sparseness (exclusion of the “rare words”) 1,490 terms were retained from the selected 983 documents. The most relevant words according to the TFIDF ponderation system (TFIDF≥5) are represented in Figure 4 as a cloud in which the size of the font is proportional to the TFIDF of every word. Looking at the first 10 word stems, the most important was calv- (TFIDF=18.4), followed by transport (TFIDF=13.8), product (TFIDF=12.3), slaughter (TFIDF=11.6), system and farm (TFIDF=10.7), consum- (TFIDF=10.5), stun- (TFIDF=10.1), castrat- (9.9), and behaviour (9.6).

### Topic Visualisation

**Figure 5:**
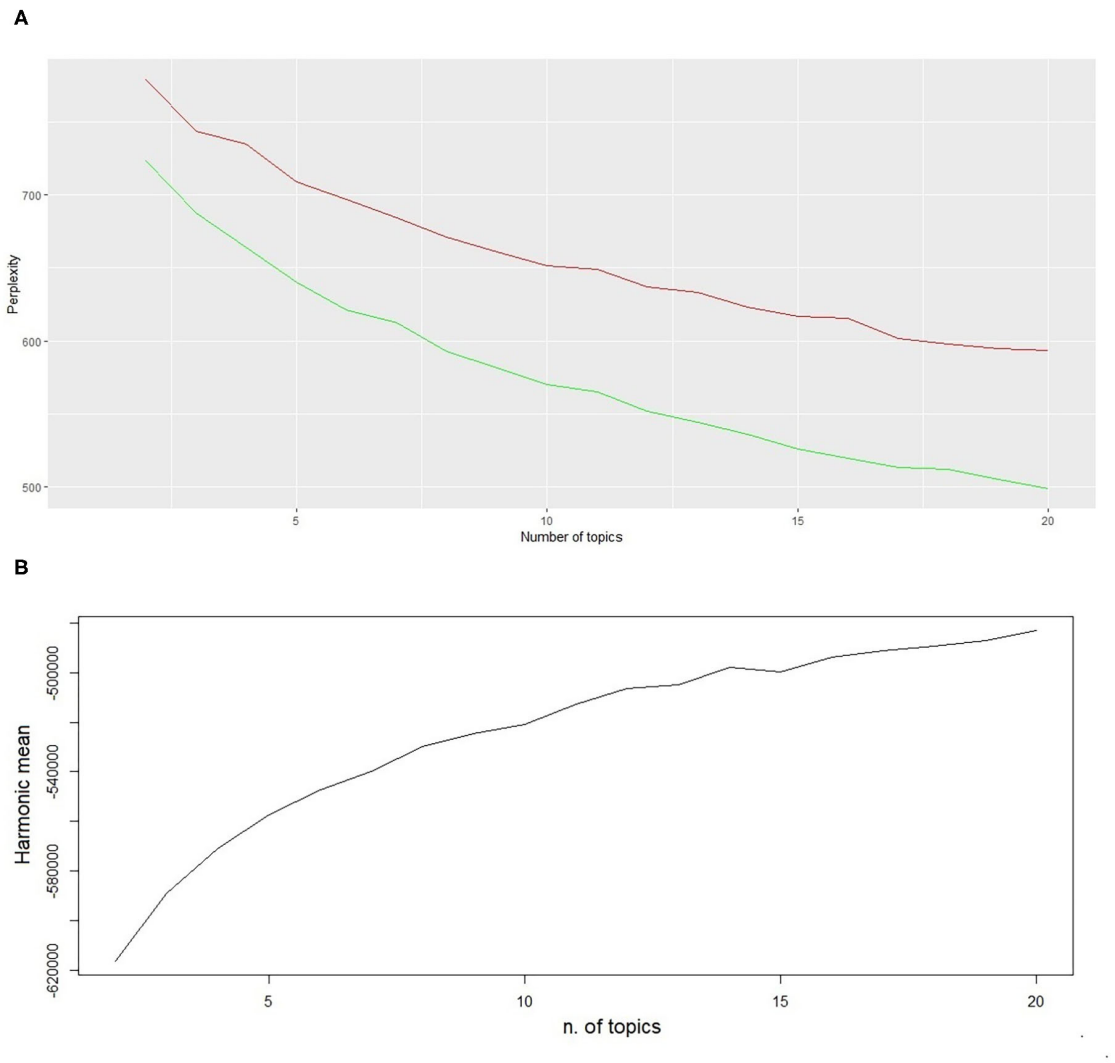
**(A)** Perplexity index of training (green) and test (red) datasets for different number of topics. **(B)** Harmonic mean of the likelihood of a set of samples generated by the Gibbs sampler.

We created a visualisation (histograms) of the ten most probable words of the topics in LDA taking into account five, ten, 15 and 20 topics, respectively. The cumulative probability of the ten most frequent words for LDA with five topics was smaller than that for LDA with ten, 15 and 20 topics. The average cumulative probability of the ten most frequent words was 0.19, 0.26, 0.31, and 0.35 for 5, 10, 15, and 20 topics, respectively. This is because if a small number of topics is assumed, a few words may not convey a topic meaning sufficiently and different issues will be lumped together (10). By expanding the number of topics assumed it is possible to discover additional themes. We tried several approaches to select an optimal number of topics. The first was based on the calculation of the perplexity index for a training and a test dataset, respectively (19). A second approach was based on the harmonic means of the likelihood of different models obtained with different number of topics (20). The results of these two approaches are shown in Figure 5.

As expected (Figure 5A), the perplexity of the training dataset was lower than the one calculated for the test set. Both curves decreased as the number of topics increased. In Figure 5B the harmonic means was instead increasing. No local minimum or maximum were found for perplexity or harmonic means. The two functions were monotonically decreasing or increasing, respectively, according to the increasing number of topics. Therefore, no clear suggestion on the ideal number of topics could be derived from these analyses.

Lastly, we considered the hierarchical clustering of the topics obtained with five, ten, 15 and 20 topics. The within-class variance component accounted for 89, 96, 96, and 97% of variance, respectively. The maximum increment was accomplished between five and ten topics; a plateau was reached with higher values. This means, in practice, that beyond ten topics there was no improvement in the capability of the model.

**Figure 6:**
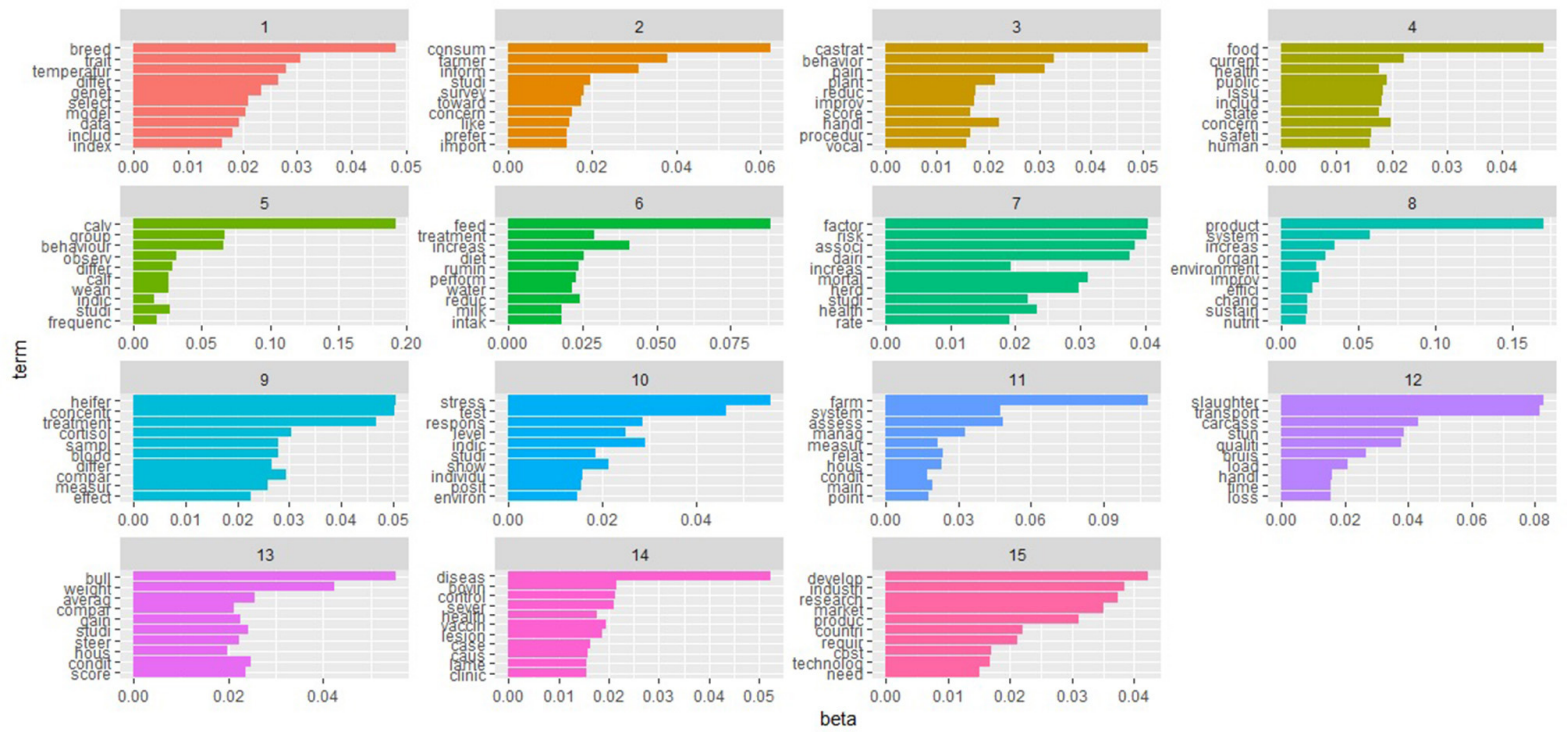
Histograms representing the most relevant words per topic in the LDA with 15 topics (beta=probability that a word belongs to a given topic).

Taking into account the outcomes of these three analyses, we selected the LDA with 15 topics. The ten most probable words in the 15 topics are reported in Figure 6.

Based on the most relevant words and the papers belonging to each topic, we tentatively attributed a theme to each topic (Table 2). The first three topics per number of papers published under each topic were topic 12 (effects of transport and slaughter on carcass quality), topic 2 (stakeholders' perceptions), and topic 8 (efficiency and environmental sustainability). Topic 4 (food safety and public health) closely followed topic 8. The cumulative probabilities (cp) calculated for the ten most relevant words in the topics showed that topics 5 (calf behaviour and management), 8 (efficiency and environmental sustainability) and 12 (effects of transport and slaughter on carcass quality) were the most important statistically (cp = 0.50, 0.41 and 0.38, respectively).

**Table 2:** Topics that emerge from the LDA analysis.

**Topic**	**Theme**	**Papers (*n*)**
1	Genetic selection and breeding	54
2	Stakeholders' perceptions	100
3	Pain management	68
4	Topics in food safety and public health	80
5	Calf behaviour and management	46
6	Feeding strategies and performance	47
7	Risk factors for morbidity and mortality	44
8	Efficiency and environmental sustainability	81
9	Health and welfare of heifers	64
10	Stress responses and indicators	48
11	Housing and management	36
12	Transport, slaughter and carcass quality	116
13	Housing conditions and weight gain in steers and bulls	61
14	Prevention and treatment of disease	66
15	New economic models	72

*We attributed a description to each topic by considering the 10 most probable words per topic in the LDA with 15 topics. The number of papers dealing with each topic is also shown*.

Table 3 shows the results of the trend analysis for the period 1990 to 2019. For each topic, the percentage of papers published in the considered time interval was analysed as a function of the year. The estimated regression coefficient represents the variation (increase/decrease depending on the sign of the coefficient) in the percentage of published papers for each incremental unit of the time. There was a significant increase in the number of papers dealing with topics 2 (stakeholders' perceptions), 7 (risk factors for health and mortality), 9 (health and welfare of heifers) and 14 (prevention and treatment of common bovine diseases), whereas the numbers of papers published on topic 15 (new economic models) decreased in the considered time frame. Topic 12 (effects of transport and slaughter on carcass quality), although the first for number of papers published over time, had a stable trend, whereas topic 2 (stakeholders' perceptions) was the one showing the most pronounced trend toward increase.

**Table 3:** Trend analysis of the 15 LDA topics by year (1990–2019).

**Topic**	**Year of appearance of first publication**	**Regression co-efficient**	***P***	***R*^**2**^**
1	1996	0.001	0.333	0.04
2	1997	0.004	0.001	0.34
3	1994	−0.002	0.426	0.02
4	1994	<-0.001	0.980	<0.01
5	1994	<-0.001	0.761	<0.01
6	1998	<-0.001	0.987	<0.01
7	2002	0.003	0.003	0.30
8	1993	<0.001	0.681	<0.01
9	1998	0.002	0.014	0.21
10	1995	−0.001	0.425	0.02
11	1993	<-0.001	0.958	<0.01
12	1990	<-0.001	0.803	<0.01
13	1993	−0.003	0.120	0.09
14	1997	0.003	0.017	0.20
15	1990	−0.006	0.020	0.20

*The regression coefficient, estimated parameter of the regression analysis, represents the incremental variation (increase/decrease) of the percentage of published scientific papers for each year. P is the significance and R^2^ is the coefficient of determination of the model*.

## Discussion

LDA applied to academic papers on beef cattle welfare captured several fields of investigation that are of known relevance, for instance pain relief during invasive procedures, the effects of transport and slaughter, the management of heifers and calves, and the treatment and prevention of disease. This characteristic—being able to identify known facts—has been proposed as evidence of the trustworthiness of the text mining algorithms (10). Additionally, the terms “heifer” and “calf” were included in the search keywords, and the topic analysis recaptured them, which is an indicator of the soundness of the methodology used. LDA modelling also revealed other topics that are of increasing societal interest, such as the link between animal welfare, sustainability and wider market issues, and the attitudes and perceptions of stakeholders such as consumers and farmers.

The geographical distribution of the first authors shows that half of the papers had first authors in Europe, followed by the United States. Besides having a strong focus on scholarly publishing in English, these two geographical areas have similar beef production data [10.64 million tonnes for the EU vs. 12.22 million tonnes for the United States; (2)]. Within the EU, the UK had the highest proportion of first authors, which does not surprise, as the UK is the cradle of the farmed animal welfare movement in Europe (21, 22) as well as being an important beef producing country. The first ten countries with high proportions of first authors are also among the main beef producing countries in Europe, with the exception of Switzerland, which however, just like the UK, has a long-standing tradition of protecting animal welfare [since 1992, Switzerland has recognised in its constitution the concept of animal dignity as well as animal well-being; (23)]. Compared to other fields of research, in which Asia (China in particular), South America and Oceania are very well-represented in terms of scientific publications, beef cattle welfare literature in English was less popular.

From 1960 to 1989, our search criteria identified on average less than one relevant paper per year published in English on beef cattle welfare. This is not surprising if we consider the history of animal welfare science. As highlighted by Broom (24), in the 1970s and early 1980s the term animal welfare was used but still not defined, and many considered it to be unscientific. Only in the early 1990s did scientists agree that animal welfare is measurable, and hence that it is a scientific concept (24). Our results show a steady increase in the number of papers on beef cattle welfare published yearly in English starting from 1996. Considering that cattle numbers worldwide increased in a gradual and consistent manner from the 1960s onwards (1), the number of published research papers on beef cattle welfare appears to have been markedly influenced by the evolution of animal welfare science.

The main word stems that emerged from the TDIF analysis were, in descending order of importance, “calv-,” “transport-,” “product-,” “slaughter,” “system-,” “farm-,” “consum-,” “stun-,” “castrat-,” and “behaviour.” Most of these terms are also included in the first three topics identified after LDA analysis, and therefore they will be discussed below.

The identified topics are comprehensive as, besides animal welfare, they include animal health and behaviour, meat quality, sustainability, and the social dimension. The analysis of cumulative probability identified the three most important topics statistically. The most significant was “calf behaviour and management” (topic 5). The tokenised words belonging to this topic were “calv-, group-, behaviour-, observ-, differ-, calf-, wean-, indic-, studi-, frequenc-.” The articles retained in the database reveal a scientific focus on aspects of animal welfare at calving and the optimisation of calf welfare (based on behavioural and physiological indicators) to reduce stress and improve growth. Some aspects were common to the literature on beef and dairy cattle, whereas others were specific to each sector. A common aspect is the correct management of difficult calving (dystocia) from the perspective of the health and welfare of both the cow and calf (25, 26). Several studies also assessed weaning stress. The weaning process includes handling, a more or less abrupt cow-calf separation and, sometimes simultaneously, transportation, sudden changes in the diet, and social reorganisation due to regrouping. All of the events taking place around this phase are sources of stress and potential health problems for both beef and dairy calves (27, 28). Cows also experience stress due to separation from their calves at weaning (28–30). Studies assessing methods to reduce stress around weaning typically rely on a mixture of behavioural and physiological indicators to compare the relative benefits of different separation strategies (e.g., two-step weaning; (27, 31, 32). Handling and transport also have an effect on stress indicators and growth in beef calves (33). The veal sector presents specific challenges: male dairy calves are typically separated from the cows immediately or 24–48 h after birth. They are normally housed individually for the first weeks of life and weaning—the gradual change in diet from milk or milk replacers to solid feed—can occur as early as 6 weeks of age. This is in itself a very stressful event with potentially long-lasting consequences (34). Research has shown that male dairy calves are given less care and attention than heifer calves due to their lower commercial value (7). One conclusion is that farmers need better information on colostrum feeding regime and pain management for these animals. Studies in the database also dealt with the management of calves kept in group housing to improve animal welfare and maximise growth (35–37).

The second most important topic identified by the statistical analysis was “efficiency and environmental sustainability” (topic 8). The word stems making up this theme are “product-, system-, increase-, organ-, environment-, improv-, effici-, chang-, sustain-, nutrit-.” The recurring theme in this group of papers is a recognition that the beef industry is under public scrutiny for several respects (e.g., food safety, environmental footprint, animal welfare) and that several adaptations will be necessary to address all of these concerns (38, 39). According to the selected literature, such adaptations can be possibly achieved by improving production efficiency and meat quality, addressing animal welfare issues, and diversifying rearing systems wherever possible, for instance by adopting organic or agro-ecological farming techniques (40, 41). Achieving and expanding the profitability of “alternative” beef rearing systems depends on a complex interplay of factors. These include market readiness and resource availability (42, 43), applied technical knowledge (41, 44), land suitability and availability (45), and whether it is possible to guarantee animal health and welfare under a range of climatic and geographical conditions (46–48). Remote sensor technology can assist in monitoring animal welfare in grazing systems (49, 50).

The third most important topic dealt with “transport, slaughter and carcass quality” (topic 12). The word stems included in this topic are “slaughter, transport, carcass-, stun-, qualiti-, bruis-, load-, handl-, time, loss-.” The identified literature reflects a tension between the economically driven pressure to increase slaughter speed on the one hand, and the need to minimise financial losses due to bruising, guarantee meat quality, and protect animal welfare and public health on the other (51). Minimising stress and suffering during the pre-slaughter and slaughter phases is an important component of overall cattle welfare, in part also due to societal expectations about how food animals should be killed (52, 53). From an economic perspective, stress and rough handling during transport, lairage and slaughter can compromise carcass quality (54–56) with consequences that can even affect the global market price of beef originating from a specific country (57). One of the most studied aspects in this group of papers concerned the effects of transport on cattle welfare and meat quality, as well as potential strategies to mitigate the associated risks (58–60). In effect, the financial losses due to rough handling during the pre-slaughter phase can be significant (61). In the slaughter phase, stun quality is important to ensure that animals do not regain consciousness until death (62); to reduce health and safety risks for slaughterhouse operators (63); to preserve meat quality, as incorrect stunning causes a surge in blood cortisol and the secretion of heat shock proteins (64). Non-stun slaughter presents specific challenges for animal welfare, such as time to loss of consciousness after the neck cut (65). Some papers investigated ante- and post-mortem animal-based indicators to assess and improve animal welfare on farm and during transport (66, 67).

Although not included in the first three most statistically relevant topics, “castrat-“ was among the first ten most important word stems according to the TFIDF analysis and is included in Topic 3 (“pain management”). The castration of male calves or mature bulls is a common practice in many parts of the world and is carried out to facilitate management and prevent unwanted breeding (68). Castration can be carried out upon arrival at the feedlot (69), in some cases together with other painful procedures [e.g., dehorning, branding (70)]. Physiological and behavioural indicators of inflammation and pain can last for days or weeks depending on the method and age of the animal at the time of castration (71, 72). The legal requirements on the provision of pain relief during castration and other painful procedures differ by geographical region and even by country. However, there is increasing awareness on this topic and veterinary codes of practice as well as some industry guidelines increasingly recommend the use of anaesthesia and/or analgesia, especially when castrating older animals [see (73–75) for some examples]. Research papers on castration included in Topic 3 focused on the availability and potential effectiveness of methods to reduce or eliminate the acute and chronic pain associated with this procedure (69, 76–78). A painless alternative such as immunisation against GnRF (gonadotropin-releasing factor) could be a viable option according to some studies (79, 80). With a view to acknowledging scientific evidence on the acute and chronic pain caused by routine invasive husbandry practices, and to meet societal expectations on the ethical treatment of farmed animals, some authors have proposed a “3S” approach (“suppress, substitute, soothe”), which is the equivalent of the “3R” (“reduce, replace, refine”) principle for animals used in research (81).

Another interesting aspect that emerged from this study is the evolution of different topics over time. Topic n. 2 (“stakeholders' perceptions”) showed the most pronounced upward trend throughout the years. This topic deals with the attitudes, beliefs, expectations and preferences of different stakeholders (citizens/consumers, veterinarians, farmers, etc.) toward animal welfare and other attributes of beef meat. Such aspects have important implications for the treatment of animals on the one hand, and for the market on the other, since they influence purchase decisions. Consumers' perceptions of the beef cattle industry—and the livestock industry at large—are constantly changing and can influence willingness to pay for meat produced and marketed in certain ways (82–84). At the same time, farmers' perceptions and beliefs can have a profound impact on their behaviour, and thus also on animal welfare (85, 86). The same applies to cattle veterinarians (87, 88) and hauliers (89).

The second topic showing the most significant upward trend in terms of papers published was n. 7 (“risk factors for morbidity and mortality”). Papers in this thematic group deal with risk factors for health and mortality in all categories of beef cattle. They are all quite recent, dating from 2002 onwards. The topics that feature most prominently in this thematic group are (a) antimicrobial use and resistance and (b) calf health, with particular reference to factors affecting morbidity and early mortality. The two topics are interdependent: veal calves are typically transported to specialised fattening facilities when still unweaned, sometimes passing via auction markets, and often with insufficient passive immunity (90). Transport over long distances, lairage and handling at auctions, and mixing upon arrival at the fattening facility are all health and welfare challenges (90, 91). As a result, morbidity, mortality and antimicrobial use are still high in the veal sector, and solutions are needed to improve the health and welfare of veal calves, also in light of the global fight against antimicrobial resistance (92–94). Other papers deal with mortality rates in cow-calf and beef fattening operations (95–97). The trend for an increase in the number of publications on these topics is very pronounced, showing a growing interest in improving animal health and welfare also as a means to protect public health.

Text mining with LDA is a methodology that enables researchers to have a good overview of the current state of a given domain or topic and provide indications for further research if relevant, especially when the number of documents to consider is large (10). However, two important limitations of our study are that the document selection was restricted to peer-reviewed research (Scopus) and to articles written in English or having at least an abstract in English. The choice of Scopus was based on the fact that it is a citational bibliometric database comprising a greater number of scientific journals compared to other databases. However, one limitation of Scopus is that it does not include grey literature, which could have been an interesting source of additional information. Concerning the literature in English, by including in our search criteria all relevant papers with at least an abstract in English, we managed to cover a broad geographical range for our research. However, as already mentioned in the results, geographical areas that normally have a good scientific output in English for other disciplines were less represented in our database. For instance, papers with a first author located in South America, where beef production is economically and numerically important, represent 11% of our database. However, due to the specific challenges associated to the rearing, handling, transport, and slaughter of beef cattle in that geographical area, it is plausible that text mining on papers written in Spanish and Portuguese, as well as in other languages (e.g., German, Chinese, etc.), will reveal different trends and topics. For this reason, an analysis of non-English literature on beef cattle welfare certainly merits to be carried out.

Based on the analysis of the top ten word stems, the most important topics statistically, and the emerging trends, two considerations can be made. The first one is that animal welfare is now perceived as an important component of beef cattle management, and one that can have a positive impact on animal and human health. This is perfectly in line with the OneHealth framework, especially concerning the global fight against antimicrobial resistance (98). The second consideration is that, based on our TM analysis of the literature in English, it would appear that research on beef cattle welfare is increasingly addressing wider societal concerns that, albeit to a variable extent, are part of contemporary global policy discussions on livestock farming. Such concerns include most notably environmental sustainability, but also production efficiency, painful husbandry procedures, as well as the attitudes of various stakeholders toward beef cattle farming.

## Conclusions

Our LDA topic analysis of scholarly articles on beef cattle welfare published in English between 1990 and 2019 shows an increasing scrutiny into the health and welfare of calves, including behavioural aspects. There is also a growing interest in sustainability issues and organic farming practices. Animal welfare during pre-slaughter and stunning, especially during transport, has an impact on meat quality and is therefore also an important research topic. In this specific case, there is a clear convergence of interest between financial gains and improved cattle welfare in the pre-slaughter and slaughter phases. The results indicate a particular focus on the welfare of calves, especially in the veal industry. Research is also increasingly assessing aspects of beef cattle welfare that are interlinked to meat quality, the social and environmental sustainability of the sector in relation to market opportunities, and public health. The issue of pain relief during castration featured prominently and is likely to become increasingly important as societal scrutiny into the ethical treatment of farmed animals converges with the scientific evidence on the acute and chronic painfulness of routine husbandry practices. The topic showing the most significant increase in popularity in the scientific literature on beef cattle welfare had to do with attitudes of consumers, farmers, and other stakeholders in the beef supply chain and their role in driving higher welfare practices and market opportunities. Another cluster of topics that has shown a marked increase in the literature since 2002 has to do with risk factors for morbidity and mortality, in particular in relation to the high use of antimicrobials in veal calf fattening facilities, which should be reduced by better addressing certain calf health and welfare issues. Although in some cases the focus is still on animal health and production parameters such as meat quality, our analysis shows that research on beef cattle welfare is increasingly incorporating and analysing environmental and societal topics that are relevant for the development of future local and global policies on livestock productions. The identified topics represent a basic source of information that can be used for further and more detailed analyses (e.g., systematic reviews) focussed on specific research themes or geographical areas.

## Data Availability Statement

The original contributions presented in the study are included in the article/Supplementary Material, further inquiries can be directed to the corresponding author/s.

## Author Contributions

EN, FG, and GC contributed to the conceptualisation and methodology of the original draft. BC performed the formal analysis. EN, BC, FG, and GC contributed to writing, reviewing, and editing the manuscript.

## Conflict of Interest

The authors declare that the research was conducted in the absence of any commercial or financial relationships that could be construed as a potential conflict of interest.
