# Supplementary material for: The Welfare of Beef Cattle in the Scientific Literature From 1990 to 2019: A Text Mining Approach
Source: Front Vet Sci. 2021 Jan 11;7:588749. doi: 10.3389/fvets.2020.588749 (PMC7832582; doi:10.3389/fvets.2020.588749)
Supplement: Supplementary file 1 [file Data_Sheet_1.pdf]

Supplementary materials

Cluster analysis on the topics

To explore the relationship between topics, we performed hierarchical clustering analysis and clustered topics based on the topic-word matrix, which was transformed to binary data with a 1/0 to indicate presence/absence of a word in a given topic. The distance among topics was calculated based on the Jaccard distance and the average linkage method was applied with an agglomerative clustering algorithm to generate the cluster dendrogram. The automatic truncation is based on the entropy and tries to create homogeneous groups. At each step of clustering process, the variability among groups is maximized and the variability within groups is minimized, consequently dissimilarity level increases. When the maximum level of entropy is reached, the aggregation process is stopped and no other steps are needed to meet a best performance of the model. The hierarchical clustering (cluster dendrogram, Supplementary Figure 1) revealed several distinct clusters of topics.

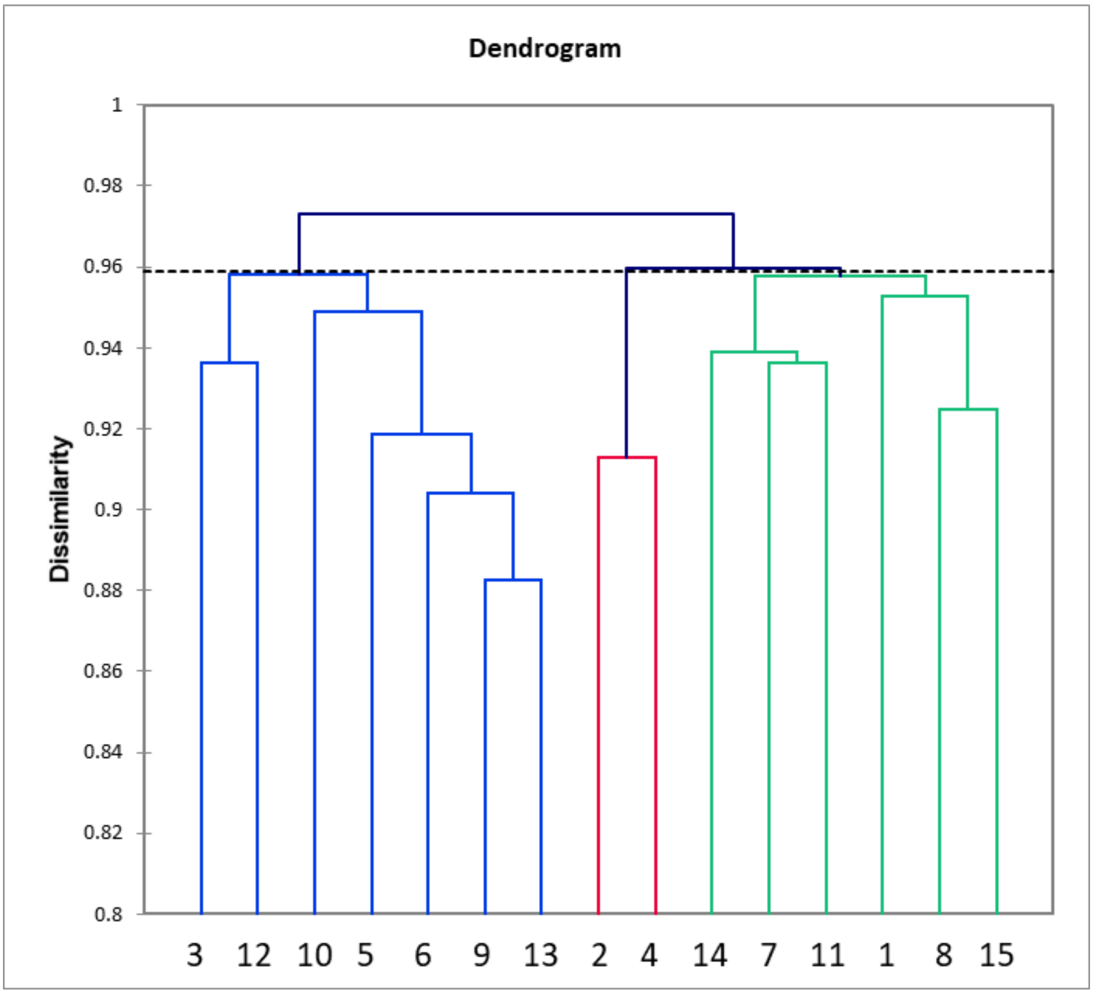

Supplementary Figure 1. Hierarchical clustering of the 15 topics identified by LDA analysis. The stippled line represents truncation and is based on a criterion of entropy.

The hierarchical clustering of topics (dendrogram) identified 5 clusters. The first (topics 3 and 12) has to do with pain management and the effects of transport and slaughter; the second (topics 10,5,6,9,13) deals with the health, welfare and feeding strategies for different categories of beef cattle. The cluster with topics 2 and 4 shows an association between aspects of public health and food safety on the one hand, and consumers' and farmers' perceptions on the other. The fourth cluster (topics 14,7,11) focuses on aspect of animal health, housing and management. The last cluster (topics 1,8, 15) deals with the economic and environmental aspects of beef cattle rearing.
